# Infants Autonomic Cardio- Respiratory Responses to Nurturing Stroking Touch Delivered by the Mother or the Father

**DOI:** 10.3389/fphys.2019.01117

**Published:** 2019-08-28

**Authors:** Martine Van Puyvelde, Laetitia Collette, An-Sofie Gorissen, Nathalie Pattyn, Francis McGlone

**Affiliations:** ^1^VIPER Research Unit, LIFE Department, Royal Military Academy, Brussels, Belgium; ^2^Experimental and Applied Psychology, Department of Psychology and Educational Sciences, Vrije Universiteit Brussel, Brussels, Belgium; ^3^Clinical and Lifespan Psychology, Department of Psychology and Educational Sciences, Vrije Universiteit Brussel, Brussels, Belgium; ^4^Cancer in Pregnancy, Department of Gynaecological Oncology, Katholieke Universiteit Leuven, Leuven, Belgium; ^5^MFYS-BLITS, Human Physiology Department, Vrije Universiteit Brussel, Brussels, Belgium; ^6^Institute of Psychology, Health and Society, University of Liverpool, Liverpool, United Kingdom; ^7^School of Natural Sciences and Psychology, Faculty of Science, Liverpool John Moores University, Liverpool, United Kingdom

**Keywords:** maternal-infant touch, paternal-infant touch, stroking touch, c-tactile afferents, respiratory sinus arrhythmia

## Abstract

The building of physiological self-regulation during bonding is a crucial developmental process based on early cardio-respiratory maturation. The mother’s role as a facilitator of this physiological maturation has been evidenced and recognized in many respects. Research in fathers, however, remains sparse which may be due to the belief that bonding is a physiological behavior reserved for a mother’s maternal instinct. In the current study we compared the impact of paternal and maternal nurturing stroking touch on infants’ physiological self-regulation in terms of respiratory sinus arrhythmia (RSA). We compared the impact of a 3-min stroking period (STROKING) with a pre-baseline (PRE-STROKING) and post-baseline (POST-STROKING) of 25 mothers and 25 fathers (unrelated to one another) on their infants, aged 4–16 weeks. We registered infant electrocardiogram (ECG) and respiration to calculate infant RR-interval (RRI), respiration rate (*f*R) and (respiratory corrected) RSA (RSA*corr*). Based on video-recordings, we analyzed the stroking speed. Infants’ RSA*corr* significantly increased during and after stroking, no matter whether touch was delivered by fathers or mothers. This effect was mediated by both heart rate (HR) and respiration. However, respiratory mediation occurred later when delivered by fathers than by mothers. Both mothers’ and fathers’ stroking speed occurred within the optimal stimulation range of c-tactile (CT) afferents, a particular class of cutaneous unmyelinated, low-threshold mechano-sensitive nerves hypothesized to be involved in inter-personal bonding. The discussion builds on the idea to mitigate fathers’ doubts about their paternal capabilities and proposes a research agenda regarding the further examination of the role of nurturing touch and its underlying mechanisms within the development of infants’ physiological self-regulation. Finally, the importance of respiratory measurements in infant physiological research is emphasized.

## Introduction

Establishing physiological and emotional bonding during the 1st months of life paves the way for one’s capacity to attach and to form intimate relationships later in life (Bowlby, 1969). This bonding process comes into being by the exchange of recurrent reciprocal cycles of sensitive responsive parent care and infant signals (Papoušek, 2007) which are known to form the source of further beneficial development. From a physiological and neuroendocrine perspective, the bonding period provides critical neuroendocrine and physiological exchanges that facilitate the development of a well-functioning stress-regulation system by epigenetic regulation of glucocorticoid receptor gene expression (e.g., Meaney, 2001; Curley and Champagne, 2016). From a social-emotional perspective, a fluent cycle of parenting is characterized by parent-infant contingency helping the infant to understand its social environment (Papoušek, 1984, 2007). When analyzing parent-infant interactions from the perspective of contingency, it was shown that fathers are able to interact with a comparable sensitive responsivity as mothers (e.g., Lynn and Sawrey, 1959; Hetherington, 1970; Biller, 1971, 1974; Block, 1973; Lynn, 1974; Maccoby and Jacklin, 1974; Sawin and Parke, 1979). Moreover, there is a growing tendency of fathers with regard to the amount of interaction with their infants. In the United Kingdom, father-infant engagement multiplied 8-fold between 1975 and 1999 (Smith, 2004) and interactions have been extended from averaging 15 min to 2 h per day (Fisher et al., 1999). As with mothers, the presence or absence of a father figure as well as the quality of father-infant interaction are playing a significant role in the development of an infant’s social-emotional behavior (Black et al., 1999; Cummings et al., 2005; Ramchandani et al., 2005; Paulson et al., 2006; Shannon et al., 2006; Bretherton, 2010).

Nevertheless, when it comes to the bonding period and caregiving responsibilities in particular, fathers are still stigmatized as ‘slow to warm up’ and shy to act. Although fathers do express an increased desire to connect emotionally with their infant (Cheng et al., 2011; Lee et al., 2013; Johnson, 2017) there is a group that persevere in a ‘gendered physiology-focused’ bias (Brady et al., 2016). These fathers view infant bonding as a physiological-based behavior that is solely the province of the mother. They report feelings of uselessness during the infant’s 1st months of life, not considering themselves being on the same plane as the mother who would have—in their opinion—the physiological advantage of having carried the infant during pregnancy and breastfeeding it postnatally (Brady et al., 2016). A foundation for this persisting bias can be found in the initial iteration of attachment theory (Bowlby, 1969). Before attachment became a multiple concept (e.g., Lamb, 1981), Bowlby (1969) sketched a monotropic parenting model with the mother as the only bonding and attachment figure for an infant.

Also in research, the mother’s role has always been emphasized with the mother-infant dyad being the main focus of study in infant interaction and development. Certainly, with regard to the development of physiological self-regulation, the current insights are mainly based on mother-infant research. These studies have shown that during close body-contact, infants benefit from a transfer of mother-infant parasympathetic regulation which aids them to mature their self-regulatory capacities (e.g., Christensson et al., 1992; Bystrova et al., 2003; Winberg, 2005; Van Puyvelde et al., 2015) that still need to develop during the 1st year of life (Bar-Haim et al., 2000). This parasympathetic transfer between mothers and infants has been ascribed to the induction of parasympathetic vasodilatation, increased blood flow, temperature (Christensson et al., 1992; Bystrova et al., 2003) and in later studies to cardiorespiratory regulation (Bloch-Salisbury et al., 2014; Van Puyvelde et al., 2015). However, although the importance of a warm ‘maternal nest’ (Winberg, 2005, p. 218) in the transfer of overall parasympathetic regulation has indisputably been demonstrated (Christensson et al., 1992; Bystrova et al., 2003; Van Puyvelde et al., 2015), this does not mean that a warm ‘paternal nest’ could not bring about similar effects.

Parasympathetic regulation is reflected at a cardio-respiratory level (Thayer and Lane, 2000, 2009) by heart rate variability (HRV). Within HRV, respiratory sinus arrhythmia (RSA) reflects the specific component related to the parasympatho-inhibitory impact on the heart, mediated by the nervus vagus (i.e., cranial nerve X) (Berntson et al., 1997). RSA is obtained by measuring ECG and respiration and calculated by the peak-valley method based on a breath-by-breath analysis in coordination with the cardiac events. In the peak-valley method, the mean difference between the longest heart period during expiration and the shortest heart period during inspiration is calculated for each respiration cycle (Grossman et al., 1990). This method is preferred in infant research (Ritz et al., 2012; Van Puyvelde et al., 2014, 2015, 2019), because of infants’ immature respiratory control (Rother et al., 1989). For instance, Ritz et al. (2012) and Van Puyvelde et al. (2015) reported that the inclusion of respiration revealed that in approximately 30% of the breaths a reliable RSA-value could not be detected which would had led to false positives at these moments.

Although research into the impact of paternal care on infants’ physiological events are sparse, a significant amount of research has been done on hormone production in parents. In fathers specifically, active playing ‘rough and tumble’ interactions have been reported to be positively correlated with oxytocin (Feldman et al., 2010) and testosterone production (Rilling and Mascaro, 2017) whereas empathy-related caring interaction behavior would be negatively correlated with testosterone (Fleming et al., 2002; Mascaro et al., 2014; Weisman et al., 2014).

The associations between oxytocin levels and paternal touch are interesting with regard to the gendered physiology-focused bias in fathers reported by Brady et al. (2016). Gentle stroking has been shown to induce oxytocin release in animals (e.g., Uvnäs-Moberg et al., 1993; Winslow et al., 2003; Okabe et al., 2015) and human infants (Matthiesen et al., 2001) and to stimulate RSA in human infants (Van Puyvelde et al., 2019). One hypothesis with regard to potential underpinnings can be found in the activity of a relatively recently characterized population of mechanosensitive unmyelinated nerves called c-tactile afferents (CTs) (Vallbo et al., 1999; Löken et al., 2009). CT afferents are found in hairy skin areas of the body and are absent in the glabrous skin (Vallbo et al., 1999; Ackerley et al., 2014b) and are preferentially responsive to gentle stroking touch within a 1–10 cm/s velocity range (Löken et al., 2009). In adults, this velocity, in comparison with slower and faster velocities, has been reported to be perceived as most pleasant (Essick et al., 1999, 2008; Löken et al., 2009). In mother-infant interaction it has been shown that mothers stroked their infants within a CT-afferent optimal stroking speed window and at CT-afferent rich body locations (Croy et al., 2016; Walker et al., 2017; Van Puyvelde et al., 2019). Moreover, with regard to the earlier comments on the importance of a ‘warm nest’ it is on interest from a neurobiological point of view that CTs are optimally responsive not just to the velocity of a nurturing touch, but also to its temperature (Ackerley et al., 2014a, 2018)—again attesting to the known value of skin-to-skin contact in regulating the infants’ (Bystrova et al., 2003) as well as adults’ (Holt-Lunstad et al., 2008) temperature.

To clarify the link between stroking touch and parasympathetic stimulation, Van Puyvelde et al. (2019) recently hypothesized that a CT afferent responsive brain network (i.e., the posterior insular cortex, mid-anterior orbitofrontal cortex, anterior cingulate cortex and amygdala) (Olausson et al., 2002; McGlone et al., 2012; Gordon et al., 2013) may overlap the zones responsible for neurovisceral integration and parasympathetic regulation in terms of RSA (Thayer and Lane, 2000, 2009). Indeed, on the level of the infant brain, gentle stroking touch evokes activity in the insular cortex of 4 weeks old infants (Tuulari et al., 2017) and 8 weeks old infants (Jönsson et al., 2018). Moreover, the insula is also involved in thermo-sensation making this modality as a submodality of touch (Craig et al., 2000). On a cardiorespiratory level, increased RSA is evoked by maternal stroking touch but not by non-stroking touch (Van Puyvelde et al., 2019) and a study by Fairhurst et al. (2014) showed decreased heart-rate responses in infants in a 10 s-window after optimal CT afferent stroking touch (i.e., a velocity of 3 cm/s but not after 0.3 or 30 cm/s).

In summary, nurturing touch has been indicated as one of the key-components in the development of self-regulatory capacities in infants (RSA), a physiological nurturing behavior that for certain fathers is reserved for a mother’s maternal instinct (i.e., gendered physiology-focused bias). Research showed that only stroking affective touch, and not non-stroking affective touch provided RSA stimulation in infants and that mothers instinctively stroked their babies at CT optimal stroking speed and locations. Therefore, in the current study we aimed at examining two research questions. Firstly, whether there is a difference between the impact of stroking affective touch of mothers versus fathers on infants’ RSA. Secondly, whether there is a difference in the stroking speed and body location that fathers and mothers chose to stroke their infant.

To examine these research questions we compared the impact of stroking touch on infant RSA from fathers with that from mothers in a pre-during-post design. We monitored infant ECG and respiration and made video recordings of the stroking activity to compare the stroking speed and body location chosen by the fathers versus mothers. In agreement with the ethical commission, we chose to not compare mother-father partners in order to avoid an atmosphere of competition within the early parent system. The participants in the father group were thus unrelated to the participants in the mother group. Also, we chose to respect the infant’s ecological environment and collected the data in the respective mothers’ or fathers’ homes.

## Materials and Methods

### Participants

The study was approved by Medical Ethics Committee of the University Hospital Of Brussels (B.U.N. 143201629352). We recruited 25 mothers and 25 fathers from prenatal classes and a private midwife’s office. The average age of the mothers was 31.45 (*SD* = 3.74; range 25–41 years) and of their infants was 10.40 weeks (*SD* = 2.63; range 6–14 weeks). The average age of the fathers was 34.53 years (*SD* = 4.16; range 29–41 years) and of their infants was 10.86 weeks (*SD* = 3.53; range 6–14). All of the infants (25 males, 25 females) were healthy full-term born babies and had an Apgar score above seven (Apgar, 1966). They had a mean birth length of 49.718 cm (*SD* = 2.42; range 42–55 cm) and mean birth weight of 3.36 kg (*SD* = 0.50; range 2.5–4.7 kg). In the mother group, one infant was excluded because of fussiness (*N* = 24) and in the father group, five infants were excluded, two because of fussiness and three because the father changed the stroking location during the experiment (*N* = 20). Within this final population, there were 10 father-boy and 10 father-girl dyads and 13 mother-boy and 11 mother-girl dyads.

### Apparatus

We made use of the BioRadio TM system (Great Lakes NeuroTechnologies Inc., Cleveland, OH, United States) to register the ECG and respiration. This system delivers synchronized registration of multiple ECG and respiration signals. The BioRadio Primary Module is a wireless acquisition system that has been used in previous infant studies (e.g., Van Puyvelde et al., 2014, 2015, 2019). The video recordings were made with a Sony Handycam type HDR-CX160. To perform the statistical analyses, we used the Statistical Package for Social Sciences Version 25.0 (SPSS 25.0). To analyze the physiological signals, we made use of VivoSense software version 3.1 (Vivonoetics, San Diego, CA, United States) and to calculate the stroking speed, we utilized the movement tracking software, Tracker 4.9.8, from the open source Physics by Brown (2018).

### Monitoring of the Physiological Signals

We registered ECG by a standard single-channel ECG registrations (II derivation) as required by standard configurations with one electrode on the upper right side and the lower left side of the chest and one grounding electrode on the upper left side of the chest. The ECG signals were recorded with a 960-Hz sampling frequency filtered by a lowpass Bessel filter order 4 with a lower cutoff of 100 Hz. For the breathing we applied a lowpass Bessel filter order 2 with a lower cutoff at 1 Hz. The breathing movements of the mother/father were measured by a thoraco-abdominal respiratory effort belt, and those of the infant with a pediatric belt.

### Questionnaires

We used the Touch Experiences and Attitudes Questionnaire (TEAQ) (Trotter et al., 2018) and the Paternal Postnatal Attitude Scale (PPAS) (Condon et al., 2008). The TEAQ inquires upon the attitudes and experiences with regard to affective touch. The TEAQ consists of six subscales, i.e., Current Social Touch (CST), Childhood Touch (ChT), Current Intimate Touch (CIT), Attitude to Intimate Touch (AIT), Attitude to Personal Grooming (APG), and the Attitude to Unfamiliar Touch (AUT). The PPAS examines the attitude of fathers toward their infant. It comprises the following components: Patience and Tolerance (PaT), Pleasure in Interaction (PiT) and Affection and Pride (AaP).

### Procedure

The data were collected during home visits. In correspondence with Van Puyvelde et al. (2019), we asked the mothers/fathers to provide a room temperature of 22–24°Celsius (Blume-Peytavi et al., 2016). After the installation of the mobile lab, undressing the infant (except diaper) and fitting the electrodes and respiration belts, the mothers/fathers were asked to sit with their infant in a manner that felt comfortable to stroke. They were requested to not place the infant on their chest to exclude a confounding of cardiac transfer (as shown in Bloch-Salisbury et al., 2014; Van Puyvelde et al., 2015) independent from a potential stroking effect.

The experimental design contained three within-subjects’ conditions, i.e., a pre-stroking baseline condition (PRE-STROKING, 3 min), a stroking period (STROKING, 3 min 20 s) and a post-stroking baseline condition (POST-STROKING, 3 min). During both PRE-STROKING and POST-STROKING, the mothers/fathers were asked to not interact with the infant. During the STROKING PERIOD, the mothers/fathers were asked to stroke their infant as they would normally do. We instructed them to stroke in a straight line but they were free to choose the body location. To avoid a potential risk on fatiguing CTs (Vallbo et al., 1999) we imposed an alternating scheme of 1 min stroking and 10 s pause, hence the STROKING duration of 3 min 20 s. The mother/father indicated when to start the experiment the PRE-STROKING condition. Afterward, the experimenter signed when to proceed to a next condition or when to stroke and when to pause. When the experiment was finished, we immediately removed the electrodes so that the parent could dress the infant again. Finally, the questionnaires were completed and a feedback was asked from the parent.

### Physiological Signal Analysis

The ECG and respiration data were visually inspected for artifacts and (in)correct detections. When ectopic beats or erroneous detections were found, the data were manually corrected (removal of erroneous detection/artifact followed by a cubic spline interpolation; corrections <1%). Other undesired events in the physiology due to sneezing, coughing, caresses during baseline conditions, rocking the infant were removed as artifacts. These moments were selected by inspecting frame by frame the video recordings. In the parents’ analysis, meanly 5.47 s per dyad was excluded which is <1% and in the infants mainly 5.93 s per dyad was excluded which is <1.1%. The timing of the detected R-wave was used to generate the RRI. For each testing block, infants’ *f*R, RRI, and RSA were calculated. RSA was computed using the peak-valley method (Grossman et al., 1990), as advised for infant research (Ritz et al., 2012; Van Puyvelde et al., 2014, 2015, 2019). The VivoSense software provides programed algorithms that correspond with the advised standards of Grossman et al. (1990, 1991). For infant ECG, the ECG RR-lockout period for R-wave picking was adjusted to 0.1. For breathing detection, the algorithm sets a minimum peak-trough value that must be exceeded for a minimum-maximum pair to be denoted as an actual breath. This helps to eliminate non-respiratory small visceral movements from being marked as breaths. This minimum value is denoted as the minimum tidal volume in the properties of the breath channel. We adjusted the default value to infant norms of 5–30 ml. In line with Grossman et al. (1990), the inspiratory and expiratory windows were moved forward 750 ms in order to accommodate the phase shifts occurring between heart period and respiration (e.g., Eckberg, 2003). The VivoSense software also accounts for violations of the Nyquist criterion (i.e., the requirement that the sampling rate is at least twice as high as the frequency of interest) by scoring the breaths with no detectable peak-valley RSA as zero.

### Data Preparation: Respiratory Controlled RSA (RSA*corr*)

Respiratory sinus arrhythmia is a multiple determined variable (Berntson et al., 2007) that reflects a combination of respiratory and somatomotor metabolic parameters (Beauchaine, 2001; Berntson et al., 2007). RSA is impacted by both respiration and speech (Tininenko et al., 2012). Therefore, it is necessary to disentangle effects of *f*R on RSA and to avoid speech during registration meant for RSA-measurement. Hence, we instructed the parents to not speak during the experiment. Further, to control for respiration effects, we applied the statistical within-subject regression approach (e.g., Grossman and Taylor, 2007) which is based on within-subject regressions on the averages of the data of each experimental block (see Grossman et al., 1991; Grossman and Taylor, 2007) with within-subjects *z*-transformations (Bush et al., 1993) as recommended in Van Puyvelde et al. (2014, 2015, 2019).

### Video-Analysis

#### Stroking Speed

To analyze the mothers’ and fathers’ stroking speed, we made use of the video recordings. The stroking periods were cut into three blocks of one minute. First, a calibration was applied. We used auto-tracking analysis when a point mass (i.e., a central recognition point on the finger of the mother/father) was recognized through the entire length of the stroking period. If the point mass was not recognized through the entire stroking period, the manual tracking function was used (i.e., a frame by frame analysis). The stroking speed is the calculated distance divided by time. For analysis, we used the stroking speed across the entire STROKING condition (Croy et al., 2016; Van Puyvelde et al., 2019).

#### Infant Motor Behavior

Based on the video recordings, we selected moments of motor activity and/or fussiness and removed them from analysis since this impacts RSA (Bazhenova et al., 2001; Ritz et al., 2012; Van Puyvelde et al., 2015, 2019). Based on the coding system of Bazhenova et al. (2001), we analyzed the motor behavioral states of the infants. Infants were in a quiet motor behavioral state for >95% of the time.

### Statistical Analysis

All of the data were tested on normality (Kolmogorov–Smirnoff test), homogeneity and sphericity. Only in case of violation details will be reported.

#### Presentation of Respiratory Uncorrected Raw Physiological

The raw data were presented based on exploratory one-way repeated measures analyses of variance (ANOVA) for each group (infants stroked by mother and infants stroked by father) with condition (PRE-STROKING, STROKING, POST-STROKING) as within subjects variables and RSA, RRI, and *f*R as dependent variables.

#### Main Analysis

The main analysis consisted of three 3 × 2 (condition [PRE-STROKING, STROKING, POST-STROKING] × group [fathers, mothers]) mixed ANOVAs with condition as within-subjects, group as between subject factor and RSA*corr*, RRI and *f*R as dependent variable to evaluate the difference in impact of stroking touch on infants’ RSA*corr*, RRI and *f*R delivered by mothers versus fathers.

#### Gender and Age Effects

Before testing for age and gender effects, we tested whether the infants in the mother versus father group were matched in age by an independent-samples *t*-test with age as dependent and group (mothers, fathers) as independent variable. Gender and age effects were tested by a series of 3 × 2 × 2 (condition [PRE-STROKING, STROKING, POST-STROKING] x group [mothers, fathers] x gender [boys, girls]) mixed ANOVAs and a 3 × 2 × 3 (condition [PRE-STROKING, STROKING, POST-STROKING] x group [mothers, fathers] x age [<8 weeks, 8–12 weeks, >12 weeks]) mixed ANOVA with condition as within-subjects factor, group and age/gender as between subject factor and RSA, RSA*corr*, RRI and *f*R as dependent variables.

#### Post hoc

For the repeated measure ANOVA tests, an extra evaluation of the proportion of variance (i.e., effect size or partial eta-squared, ηp^2^) was made. We also performed a pairwise comparison by *post hoc* tests with the critical *p-*value for significance adjusted with Bonferroni correction. The confidence interval calculation was based on the within-subjects approach for repeated measures of Cousineau (2005). The method of Cousineau (2005) normalizes confidence intervals for within-subject designs.

#### Stroking Speed

A one-sample *t*-test compared the velocity rate of fathers and mothers with the mean velocity rate stroking speed observed in Croy et al. (2016).

#### Body Locations

The video recordings were also analyzed on the chosen body locations by both mothers and fathers.

#### Questionnaires

An independent-samples *t*-test tested the difference in scores self-reported by mothers and fathers on the TEAQ. A one-sample *t*-test compared the scores of the fathers on the PPAS in the current study with the mean scores reported in Condon et al. (2008).

## Results

### Presentation of Respiratory Uncorrected Raw Physiological Data

Table 1 shows an overview of the raw data and one-way ANOVA (PRE-STROKING, STROKING, and POST-STROKING) tests on respiratory uncorrected RSA, RRI, and *f*R in the infants stroked by the mothers versus the infants stroked by the fathers. We inspected the data for outliers based on the *z*-standardizations (*z* = ± 2.58). We excluded one infant in the group stroked by the mother having *z*-values > 2.56 (*N* = 23). The raw results show that there is an impact of stroking in both the mother and father group (see Table 1).

**TABLE 1:** Means (SD) and analyses using one-way repeated measures ANOVA of raw RSA values (in ms), raw RRI values (in s), and raw fR values (respiration rate, cycles per minute) during PRE-TOUCH, TOUCH, and POST-TOUCH of the infants stroked by fathers versus infants stroked by mothers.

		**PRE-TOUCH**	**TOUCH**	**POST-TOUCH**					
		***M (SD)***	***M (SD)***	***M (SD)***	***df***	***F***	***p***	**ηp^2^**	**Bonferroni**
Infants in Father Group	RSA	7.900	8.986	10.132	2, 38	7.377	**0.002^∗^**	0.280	Post > Touch, *p* = 0.016
	raw	(1.668)	(1.081)	(1.674)					
	RRI	0.404	0.406	0.415	2, 38	2.493	0.096	0.116	/
		(0.014)	(0.013)	(0.016)					
	*f*R	53.73	53.05	49.87	2,	3.618	**0.036^∗^**	0.160	Post < Touch, *p* = 0.036
		(4.74)	(3.22)	(3.73)	38				
Infants in Mother Group	RSA	8.879	10.503	11.575	2, 44	12.598	**< 0.001^∗^**	0.364	Pre < Touch, *p* = 0.027
	raw	(1.311)	(1.728)	(1.422)					Pre < Post, *p* < 0.001
	RRI	0.427	0.423	0.439	2,	6.467	**0.003^∗^**	0.219	Touch < Post, *p* = 0.003
		(0.014)	(0.010)	(0.014)	46				
	*f*R	43.80	41.33	39.56	2,	7.418	**0.002^∗^**	0.244	Pre > Touch, *p*, = 0.026;
		(3.51)	(2.45)	(3.31)	46				Touch > Post, *p* = 0.010

*Significant *p-*values are indicated in boldface with an asterisk (^∗^).*

### Main Analysis

**FIGURE 1:**
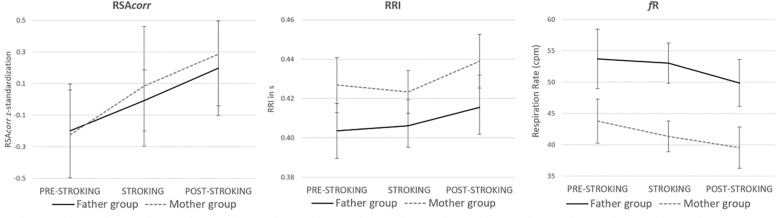
Infants’ RSA*corr*, RRI and *f*R before, during and after stroking touch in the Father group and Mother group. There was a significant main effect in RSA*corr*, RRI and *f*R and no difference between both groups. RSA significantly increased during and after stroking touch, RRI significantly increased after stroking touch and *f*R significantly decreased after stroking touch.

There was a main effect of stroking touch on infants’ RSA*corr, F*(2,82) = 16.052, *p* < 0.001, ηp^2^ = 0.281. RSA*corr* significantly increased from PRE-STROKING to STROKING (*p* = 0.007, Bonferroni corrected) and further to POST-STROKING (*p* < 0.001, Bonferroni corrected). There was no significant difference between the impact of stroking touch of mothers versus fathers (or no interaction effect condition-group) *F*(2,82) = 0.342, *p* = 0.712, ηp^2^ = 0.008. The elongated RSA*corr* main effect in POST-STROKING was mediated by both RRI and *f*R. That is, from STROKING to POST-STROKING, there was a significant main effect of stroking touch on infants’ RRI, *F*(2,84) = 7.831, *p* = 0.001, ηp^2^ = 0.157, confirmed by Bonferroni (*p* = *0.003*) with no difference between mothers and fathers (or no interaction effect condition-group), *F*(2,84) = 0.487, *p* = 0.616, ηp^2^ = 0.011 and a significant main effect on infants’ *f*R, *F*(2,84) = 9.751, *p* < 0.001, ηp^2^ = 0.188, confirmed by Bonferroni (*p* = *0.005*) with no difference between mothers and fathers (or no interaction effect condition-group), *F*(2,84) = 0.522, *p* = 0.595, ηp^2^ = 0.012 (see Figure 1).

### Gender and Age Effects

There was no significant difference in age between the infants in the father group (*M* = 10.52 weeks; *SD* = 2.67) versus the infants in the mother group (*M* = 9.99 weeks; *SD* = 2.28), *t*(42) = 0.714, *p* = 0.479. We also found no age effects on RSA*corr*, RRI or *f*R and one marginal gender effect on RRI, *F*(2,84) = 2.918, *p* = 0.059, ηp^2^ = 0.061 with a significant contrast from PRE-STROKING to POST-STROKING, *p* = 0.32 that we want to report. This contrast showed that infant boys in the father group had a larger increase in RRI from PRE-STROKING to POST-STROKING than the girls in the father group and the boys and girls in the mother group.

### Stroking Speed

In the father recordings, the point mass was not always visible, hence video-analyses were based on 23 mother and 12 father recordings. There was no significant difference between the stroking speed of mothers (*M* = 7.91; *SD* = 2.98) and fathers (*M* = 7.10; *SD* = 3.16), *t*(33) = 0.761, *p* = 0.452. The stroking speed in the current study (*M* = 7.38 cm/s; *SD* = 2.99) was slightly slower than in Croy et al. (2016) (*M* = 8.4 cm/s), *t*(34) = −2.028, *p* = 0.050.

#### Body Location

Both mothers and fathers chose body locations that are known to contain CT afferents, that is head-region, body-region and the limbs (see Table 2).

**TABLE 2:** Overview of the chosen body-locations by mothers and fathers to stroke their infant.

	**Number of mothers**	**Number of fathers**
Head-region	13	9
Body-region (back, shoulders)	5	5
Limbs	6	6

### Questionnaires

Normality was violated for one subscale of the PPAS, i.e., Affection and Pride, *D*(24) = 0.23, *p* = 0.002. Hence, no results will be interpreted with regard to this subscale. With regard to the TEAQ, fathers scored significantly lower than mothers on the items related to APG, *t*(40.25) = −2.808, *p* = 0.010. We found no correlations between the TEAQ-items and infant physiological variables. The fathers in the current study (*M* = 73.4; *SD* = 10.2) scored lower on the PPAS than in Condon et al. (2008) [*M* = 79.2; *SD* = 9.0), *t*(18) = −2.467, *p* = 0.024] due to a significant lower score on the items related to Patience and Tolerance, *t*(18) = −3.378, *p* = 0.003. We found no correlations between the PPAS-items and infant physiological variables.

## Discussion

In the current study we compared the impact of paternal and maternal affective stroking touch on the RSA, RRI and *f*R of their infants. We replicated the experimental pre-during-post design of Van Puyvelde et al. (2019) with infant ECG and respiration monitoring and simultaneous video recording of the parents’ stroking activity. In order to avoid an atmosphere of competition between parents we opted to study fathers and mothers who were unrelated to one another. Our current results showed no significant difference between the impact of paternal and maternal affective touch on our measures. When corrected for respiration, the infants’ RSA*corr* significantly increased during and after they were touched, no matter whether the touch was delivered by fathers or mothers. This shows that parental affective touch has a beneficial impact on the parasympathetic regulation, be it delivered by mothers or fathers. Moreover, in line with the mother data in Croy et al. (2016) and Van Puyvelde et al. (2019), both the mothers and the fathers in the current study stroked their infants instinctively within a CT-afferent optimal velocity range of 1–10 cm/s with an average of 7–8 cm/s and chose body locations that are known to be rich of CT afferents such as the arms, legs, shoulders and head.

The first research question has been answered affirmatively: there was no difference between the impact of stroking touch delivered by mothers with whom infants already shared a prenatal physiological history versus fathers with whom the infants did not share a prenatal physiological history. Already in the 1970’s, a series of studies showed that fathers interacted with a similar sensitivity toward their infants as mothers (e.g., Lynn and Sawrey, 1959; Hetherington, 1970; Biller, 1971, 1974; Block, 1973; Lynn, 1974; Maccoby and Jacklin, 1974; Sawin and Parke, 1979). Nevertheless, until recently (Bretherton, 2010; Brady et al., 2016), a persistent idea that giving comfort and care is the province of the mother remains. Bretherton (2010) pointed out that whatever the type of interaction parents chose to have with their infants, it is important to override doubts and to appreciate one another’s actions. Therefore, the current study provides evidence that should obviate fathers’ doubts upon the fact that they are able to evoke physiological regulatory processes in their infants during the 1st weeks of life.

The second research question has also been answered affirmatively (although with caution since only half of the father video recordings could be analyzed): there was no difference between the stroking velocity of mothers and fathers. Moreover, in line with earlier studies (Croy et al., 2016; Van Puyvelde et al., 2019), the stroking speed of both the analyzed fathers and mothers was within the optimal CT afferent stimulation window and the chosen locations of both fathers and mothers were CT afferent rich body locations (i.e., shoulders, arms, legs, head). These results give scope to discussion with regard to underlying mechanisms. The observations with regard to the stroking speed in both mothers and fathers may suggest that it was the stimulation of CT afferents that underpinned the infants’ parasympatho-inhibitory regulation, as was suggested in Van Puyvelde et al. (2019). Another clarification could be found in a simple time-effect, that is that RSA would have increased as a result of the time being together with the father. However, in mothers, a time-effect was excluded by Van Puyvelde et al. (2019) since non-stroked infants’ RSA decreased after the termination of static touch. Hence, we doubt that similar maternal and paternal touch patterns with similar impacts on infants’ RSA would result from two different underlying mechanisms. However, it is plausible that infants need some familiar social cues to identify themselves with affective touch as stated by Pirazzoli et al. (2019) and those cues were present in both the fathers and the mothers arms. To further examine the role of underlying mechanisms and influencing factors with regard to touch-related parasympathetic infant regulation, two aspects are on the research agenda. Firstly, different types of touch-providers should participate, that is strangers as well as family members in atypical family constellations such as same-gender parents and adoption parents. There is a lack of research that determines the aspects that are important to build the needed physiological and affective conditioning processes in a context different to the common biological mother-infant interaction. The current study showed that a shared prenatal context is not a critical condition to help an infant in the stimulation of parasympatho-inhibitory regulation. Hence, this study may open doors toward new research that examines parasympatho-inhibitory transfer processes within other parent-infant constellations. Secondly, to pinpoint the mechanism of CT afferents, standardized velocity rates should be compared as in Fairhurst et al. (2014) (however in longer windows in order to allow RSA-analysis on top of an acute phasic response in HR) as well as the effect of the temperature of the delivered touch—bearing in mind CTs are velocity tuned and, interestingly, their firing rate varies depending on the temperature of a touch (see Ackerley et al., 2018). Finally, all of these avenues of research should be investigated in an ecological as well as non-ecological context. The comparison with a non-ecological context could increase insight in what manner the conditioned aspect of the ecological nurturing context is important in an eventual developmental sensitization process of CT afferents. For instance, in a study by Pirazzoli et al. (2019), a brain response to affective touch in 5-months old infants could not be found, which the authors ascribed to the strict laboratory settings that were applied in place of a more natural mother-infant context.

Parasympathetic regulation was measured by registering both ECG and respiration of the infants. This remains the only way to have full insight into the cardiorespiratory system. Besides parasympathetic activity, respiratory and somato-motor metabolic parameters (Beauchaine, 2001) need to be taken into account when it comes to final interpretation. However, the real interconnectedness between heart rate (HR) and respiration, which is in fact the base of RSA, remains unknown when analyses are based on HR only (Grossman et al., 1990). Our analyses showed that the responses to parental touch in the infants were mediated by both their HR and respiration. There was a combined significant increase in RRI and decrease in *f*R. During POST-STROKING, in both the father and mother group, the infants’ heart period and respiration were engaged in what can be called a reciprocally parasympathetic activation (Berntson et al., 1991). An autonomic reactivity response often yields a combined respiratory induced sympathetic deactivation with a cardio induced parasympathetic activation (Berntson et al., 1991). The delayed reciprocally parasympathetic activation during POST-STROKING in the current study may point to the fact that affective touch contributes not only to an acute phasic reactivity response but also to the installation of a more integrated tonic response of long-term regulation. Further research needs to establish whether this delayed slower reactivity response might be the expression of the mechanism that underpins the earlier suggested role of touch within the development of an optimally functioning stress regulation system (e.g., Levine, 2001; Meaney, 2001; Champagne et al., 2008; Uchida et al., 2010; Franklin et al., 2011; Walker and McGlone, 2013; Curley and Champagne, 2016; Van Puyvelde et al., 2019).

The inclusion of respiration measures also revealed an interesting difference between the infants in the mother versus father group. In the mother group, a respiratory induced effect occurred already during the STROKING period whereas in the father group this effect was delayed and only observed during POST-STROKING. In the mother group, during the STROKING period, increased RSA occurred in combination with decreased *f*R whereas the decreased *f*R in response to touch delivered by fathers only occurred in the POST-STROKING period. Moreover, the infants in the father group had throughout the three conditions a significantly higher *f*R and RRI than those in the mother group. These differences were not due to a difference in age since the infants were matched in age. Moreover, no age-effects were found. Previous studies reported that mothers interact in a more nurturing and fathers in a more playful and stimulating manner with their infants (e.g., Rendina and Dickerscheid, 1976; Lamb, 1977, 1981; Feldman, 2003; Kokkinaki and Vasdekis, 2014). From a physiological perspective, it has been suggested that with these different interactive arousal patterns, mothers offer their infant the practice of affective regulatory coordination (Feldman, 2003) whereas fathers would offer them a manner to cope with high-intensity activity in a positive arousal state, increasing the infant’s stress resilience (Pedersen et al., 1979). In the current study we did not collect information on the amount and type of interaction the fathers and mothers respectively maintained and whether a similar distinction in interactive behavior might have caused different anticipatory physiological states in the infant. Therefore, to find out whether the difference between infants’ *f*R and RRI in the mother group versus father group was due to a coincidental interindividual variance or not, a baseline study should be conducted comparing a baseline measured independent from the respective parent with a baseline measured in the arms of the father and the mother.

Finally, we found a tendency for a beneficial impact of a father-son gender-match with regard to RRI from STROKING to POST-STROKING. Although only the contrast was significant, we want to mention this result since it corresponds with earlier behavioral observation research that reported higher levels of matched synchrony (Feldman, 2003), emotional sharing and physical proximity (Lamb, 1977, 1981; Weinraub and Frankel, 1977) in gender-matched parent-infant interactions than in non-gender-matched interactions which had been ascribed in former research to a shared biological preparedness between matched genders (Feldman, 2003).

This research had some strengths and limitations. This is one of the few studies that investigated the impact of paternal behavior on infants’ physiology and that respected the infant’s ecological home-situation. It offered a video-controlled analysis and included respiration to interpret RSA and correct it for respiratory confounds. A limitation of this time-consuming approach is a rather low number of participants. Further, a non-stroking father group, a stranger stroking and stranger non-stroking group would had been beneficial to further increase insight in the underlying mechanisms such as CT-afferent activity and the interaction with the ecological context of the infant. Finally, information on the parent-infant interactions at home would had informed on gender-typical interaction patterns in the participants.

In summary, the current study found that there is no difference between the final impact of stroking touch delivered by mothers versus fathers on the RSA*corr* of infants. Parental touch, be it delivered by a mother or a father induces parasympatho-inhibitory regulation in the infant. This regulation is mediated by both HR and respiration. However, when delivered by fathers, the respiratory effect occurred in a later stage than in mothers, i.e., in the father group after touch delivery in place of during touch. Affective touch is a gender-neutral facilitator to create a ‘parental nest’ of parasympatho-inhibitory regulation. By getting in touch through bonding, both father and mother pave the way to keep in touch through attachment.

## Data Availability

The datasets generated for this study will not be made publicly available. Currently this is not standardly included in our ethical procedures.

## Ethics Statement

This study was carried out in accordance with the recommendations of the ICH-GCP. The protocol was approved by the Medical Ethics Committee of the UZ Brussel/VUB. All subjects gave written informed consent in accordance with the Declaration of Helsinki. Our data will not be made available in accordance with the ethical procedures of ICH-GCP and GDPR.

## Author Contributions

MVP: supervision, analysis, and writing. LC: data collection and analysis. A-SF: analysis. NP: reflecting team. FM: supervision and manuscript revision.

## Conflict of Interest Statement

The authors declare that the research was conducted in the absence of any commercial or financial relationships that could be construed as a potential conflict of interest. The reviewer RA declared a past co-authorship with one of the authors FM to the handling Editor.
